# Hoisting-Loop in Bacterial Multidrug Exporter AcrB Is a Highly Flexible Hinge That Enables the Large Motion of the Subdomains

**DOI:** 10.3389/fmicb.2017.02095

**Published:** 2017-10-25

**Authors:** Martijn Zwama, Katsuhiko Hayashi, Keisuke Sakurai, Ryosuke Nakashima, Kimie Kitagawa, Kunihiko Nishino, Akihito Yamaguchi

**Affiliations:** ^1^Department of Cell Membrane Structural Biology, Institute of Scientific and Industrial Research, Osaka University, Osaka, Japan; ^2^Department of Biomolecular Science and Regulation, Institute of Scientific and Industrial Research, Osaka University, Osaka, Japan; ^3^Graduate School of Pharmaceutical Sciences, Osaka University, Osaka, Japan

**Keywords:** multidrug resistance, antimicrobial resistance, RND, AcrB, mechanism, pathogens, transporters, crystal structure

## Abstract

The overexpression of RND-type exporters is one of the main causes of multidrug resistance (MDR) in Gram-negative pathogens. In RND transporters, such as *Escherichia coli*'s main efflux pump AcrB, drug efflux occurs in the porter domain, while protons flow through the transmembrane domain: remote conformational coupling. At the border of a transmembrane helix (TM8) and subdomain PC2, there is a loop which makes a hoisting movement by a random-coil-to-α-helix change, and opens and closes a drug channel entrance. This loop is supposed to play a key role in the allosteric conformational coupling between the transmembrane and porter domain. Here we show the results of a series of flexibility loop-mutants of AcrB. We determined the crystal structure of a three amino acid truncated loop mutant, which is still a functional transporter, and show that the short α-helix between Cβ15 and the loop unwinds to a random coil in the access and binding monomers and in the extrusion monomer it makes a partially stretched coil-to-helix change. The loop has undergone compensatory conformational changes and still facilitates the opening and closing of the channel. In addition, more flexible mutated loops (proline mutated and significantly elongated) can still function during export. The flexibility in this region is however limited, as an even more truncated mutant (six amino acid deletion) becomes mostly inactive. We found that the hoisting-loop is a highly flexible hinge that enables the conformational energy transmission passively.

## Introduction

Multidrug resistance (MDR) is a serious problem in global health we face today (World Health Organization, 2014). Antimicrobial resistance in gram-negative bacterial pathogens is one of the main challenges (Poole, 2004; Li et al., 2015). The overexpression of tripartite efflux systems is a major cause of MDR in these gram-negative pathogens (Poole et al., 1993; Nikaido, 1998; Blair et al., 2014). These complexes can facilitate efflux directly across the outer membrane as they consist of a Resistance-Nodulation-Division (RND) efflux pump and an outer membrane protein, bridged together by an adapter protein (Zgurskaya and Nikaido, 2000; Lomovskaya and Totrov, 2005). In *Escherichia coli*, the main RND transporter is AcrB, part of the AcrAB-TolC complex (Nishino and Yamaguchi, 2001). This secondary efflux pump facilitates efflux by using the electrochemical proton gradient (Zgurskaya and Nikaido, 1999) and besides being the main cause of MDR when overexpressed, its physiological function in bacterial cells is to export naturally occurring toxic compounds, such as bile salts (Thanassi et al., 1997; Nishino et al., 2009). RND transporters also play a major role in virulence and in MDR in other organisms, such as the MexAB-OprM efflux system in *Pseudomonas aeruginosa*. Overexpression of RND transporters cause MDR as they recognize and transport a wide range of structurally unrelated compounds, ranging from dyes to antibiotics and from small compounds to large compounds (Nikaido and Pagès, 2012). The physiologically active form of AcrB is a homotrimer consisting of three protomers, each representing one step in the drug-extrusion cycle. This functionally rotating mechanism is shown by each of the three monomers in the asymmetrical crystal structure of AcrB: access, binding and extrusion (Murakami et al., 2006; Seeger et al., 2006). During this cycle, conformational changes take place in both the transmembrane (TM) and the porter region, which are about 50Å apart. These regions therefore have to be energy-coupled with each other in a remote conformational way.

In the transmembrane domain, several helix bundles and amino acids change their conformation upon protonation and deprotonation of crucial amino acids and the interaction with the conformational changes in the porter domain of AcrB. In the binding monomer, Asp407 is exposed to the periplasm by a water-void and a proton can bind. Now the transition to the extrusion state can occur. During this transition from the binding to the extrusion state, six N-terminal TM-helices (TM1-TM6) and six C-terminal TM-helices (TM7-TM12) are twisted relatively to each other as a result of the ion-pair breakage between Asp407, Asp408 and Lys940. During this transition to the extrusion monomer, residue Arg971 extends and bends downwards to TM10 and the phenyl-ring of Phe948 is tilted downwards and Met970 bent away. In this way, Arg971 is exposed to a water-void open to the cytoplasm and can release a proton. Now, in the extrusion monomer, the loop at the N-terminal end of TM8 performed a hoisting-like motion by its conformational change from a random coil to an α-helix (to make an extended TM8), presumably playing a key role in conformational transmission and facilitating the motion of PN1/PC2 during the transition to the extrusion monomer (Eicher et al., 2012; Yamaguchi et al., 2015).

**Figure 1:**
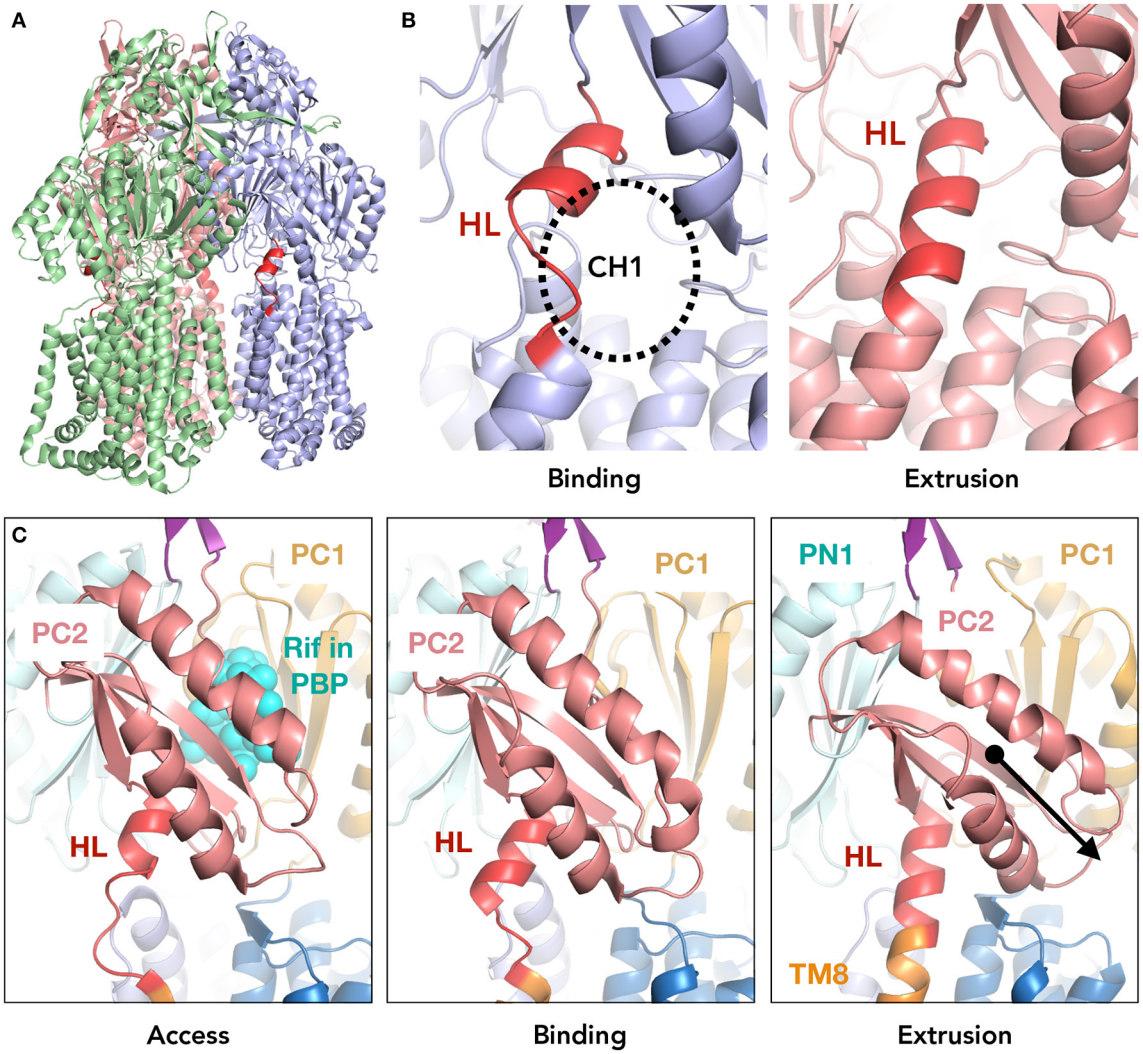
The location of the hoisting-loop in the AcrB drug transporter. **(A)** A side view of the AcrB trimer. Each protomer representing one step in the drug extrusion cycle is depicted in green, blue and light red for access, binding and extrusion, respectively. The hoisting-loop is shown in dark red. **(B)** A close-up view of the hoisting-loop in the binding and extrusion monomers. Dark red indicated the residues ^860^TGMSYQERLSGNQ^872^. The open and closed channel 1 (CH1) can be seen. **(C)** A close-up of the hoisting-loop region including TM8 and PC2 for the access, binding and extrusion monomers of AcrB. In the extrusion monomer, the PC2 domain (light red) is shifted downwards significantly along with the elongation of TM8 (altering the hoisting-loop from a random coil to an α-helix). Abbreviations: TM8, transmembrane 8; HL, hoisting-loop; CH1, channel 1; Rif, rifampicin; PBP, proximal binding pocket.

At the interface of the transmembrane and porter domain, this most significant conformational change during this extrusion cycle occurs at the region between the β-strand (Cβ15) of the PC2 subdomain and TM8 (Figure 1). This segment comprises 13 amino acid residues (^860^TGMSYQERLSGNQ^872^). In the extrusion monomer, this segment forms three turns of the α-helix which extends the top of TM8. On the other hand, in the access and binding monomer, a part of this helix (^868^LSGNQ^872^) unwinds to a random coil, enabling the upward swinging motion of PC2 by extending the distance from TM8 to Cβ5. This random-coil-to-α-helix change also opens and closes the membrane surface channel (CH1) during the drug extrusion cycle (Murakami et al., 2006; Figure 1B). Considering the location of the loop and the significant conformational change during the export cycle, the loop is assumed to be implicated in the mechanism of the energy transduction in AcrB (Murakami et al., 2006; Su et al., 2006; Sennhauser et al., 2007; Seeger et al., 2008; Pos, 2009; Eicher et al., 2012, 2014; Long et al., 2012; Yamane et al., 2013; Yu et al., 2013; Du et al., 2015; Yamaguchi et al., 2015). We created a series of loop-flexibility mutants of AcrB, ranging from low flexibility (truncated mutants) to high flexibility [proline (which break the helix) and significantly elongated (flexible gly/ser linkers) mutants] and checked the export activity of all of these mutants. Additionally, we solved the crystal structure of a truncated mutant at 3.0Å to elucidate the role of this segment of AcrB and to better understand the remote conformation coupling mechanism of RND multidrug exporters.

## Materials and methods

### Bacterial strains and growth conditions

The *E. coli* MG1655 strain (Blattner et al., 1997) was used as the wild-type strain in this study. The Δ*acrB* (NKE96) (Nishino et al., 2008) and Δ*tolC* (NKE95) (Horiyama and Nishino, 2014) deleted mutants were derived from MG1655. Gene deletion was performed according to the method of Datsenko and Wanner, with recombination between short homologous DNA regions catalyzed by phage λ Red recombinase (Datsenko and Wanner, 2000). The drug resistance markers were eliminated using plasmid pCP20, as previously described (Datsenko and Wanner, 2000). The Bacterial strains were grown at 37°C in Luria-Bertani broth (Sambrook et al., 1989).

### Drug susceptibility by MIC

The minimal inhibitory concentrations (MIC) for wild-type and mutant AcrB were determined using LB-agar plates supplemented with substrates. The substrates were added in a series of dilutions. Agar plates were stamped with the desired strains and grown at 37°C overnight. The MIC values are the concentration of drugs in the agar plates at which no cells were viable anymore. For growth curves, cells were grown from diluted exponentially growing cells in 96-well plates for 8 h at 37°C. OD_600nm_ readings were performed by using the Infinite M200 Pro (Tecan).

### Drug exclusion assay

For ethidium bromide exclusion efflux determination, BL21Δ*acrB E. coli* cells were used. Overnight cultures of the *E. coli* cells were diluted and grown at 37°C until OD_600nm_ 0.5–0.6 was reached. Cells were harvested and washed twice with Efflux Buffer (100 mM potassium phosphate (pH 7.5) and 5 mM MgSO_4_) and diluted to final OD_600nm_ of 18. 10 μM of ethidium bromide and 10 mM arabinose was added. Ethidium bromide fluorescence was measured by SH-8100 reader (Corona Electric Co.) using λ_ex_ = 530 nm and λ_em_ = 600 nm. Exclusion assays were repeated at least four times providing the error envelopes.

### Protein expression and crystallization

C-terminally His6-tagged mutant AcrB (hoisting-loop truncated mutant) were expressed in JM109Δ*acrB E. coli* cells harboring the pAcBH plasmid. The final mutant AcrB buffer condition was 20 mM sodium phosphate pH6.2, 10% (v/v) glycerol, 0.05% (w/v) n-Dodecyl-β-D-maltoside and the final protein concentration was 20 mg/ml. Crystals of AcrB variants were made by the hanging drop vapor diffusion method at 25°C. The protein solution was mixed with an equal volume of reservoir solution. The reservoir solution contained 100 mM SPG (succinic acid, sodium phosphate monobasic monohydrate and glycine) buffer at pH5.0 and 20% (w/v) PEG1500.

### Crystallographic analysis

X-ray diffraction data were collected at 100 K on beamline BL44XU at SPring-8 (Hyogo, Japan). X-ray data set was indexed, integrated and scaled using *iMosflm* (Battye et al., 2011) and *Scala* (Evans, 2006) from *CCP4* program suite (Lebedev et al., 2012). The initial structure was solved by molecular replacement using *MOLREP* (Vagin and Teplyakov, 2000) with wild-type AcrB (3AOA with manual truncation of hoisting-loop) as a search model. The model refinement was performed through multiple cycles of manual rebuilding using the program *COOT* (Emsley et al., 2010) and refinement using *REFMAC5* (Murshudov et al., 2011) with jelly-body option.

## Results

### Truncated hoisting-loop

**Figure 2:**
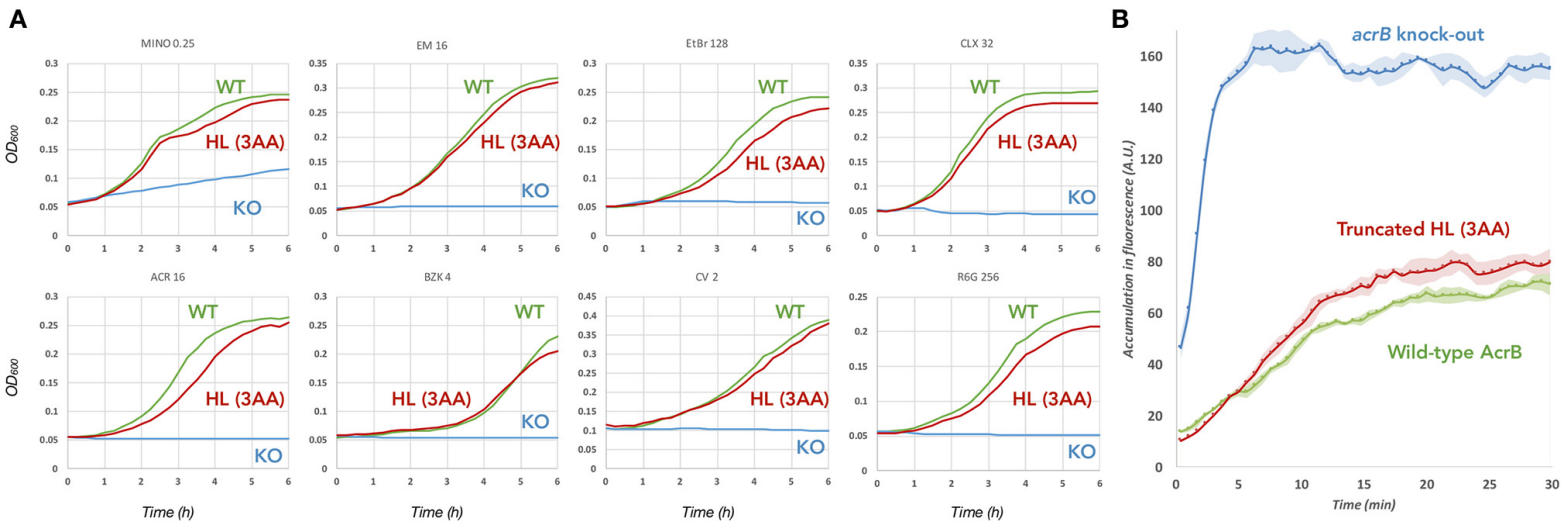
Efflux ability of the 3 amino acid truncated hoisting-loop mutant of AcrB. **(A)** Growth curves of wild-type (green) and mutant (red) AcrB expressing *E. coli* cells, compared to *acrb*-knockout cells (blue). The concentration of the drugs is written above each panel (in μg/mL) and a complete growth curve set for other concentrations can be found in Supplementary Figure 2. Shown is one of the results, repeats of the experiment gave similar results. **(B)** The ethidium bromide exclusion ability of the truncated mutant (red) compared to wild-type AcrB (green) and *acrb*-knock-out (blue). Exclusion assays were repeated at least four times providing the ± standard deviations shown by the error-envelopes. Abbreviations: WT, wild-type; KO, knock-out; HL (3AA), hoisting-loop 3 amino acids deleted mutant.

To check the role of the hoisting-loop segment, ^860^TGMSYQERLSGNQ^872^ located at the N-terminal end of TM8 (Figure 1), we created a shortened (truncated) hoisting-loop mutant by deleting the three amino acids Leu868, Asn871 and Gln872 (one turn of the α-helix, **Figure 4**). We tested the efflux ability of the mutated pump by determining the growth ability of AcrB-expressing *E. coli* cells (both wild-type and mutant) in the presence of various structurally unrelated compounds: minocycline, erythromycin, ethidium bromide, cloxacillin, acriflavine, benzalkonium, crystal violet and rhodamine 6G. We also tested the truncated mutant on ethidium bromide exclusion ability in *E. coli* cells, compared to wild-type AcrB expressing and *acrB*-knockout cells. When ethidium enters the cell, it binds to DNA and becomes fluorescent. Active AcrB transporters export ethidium out of the cell from the periplasm or outer leaflet of the inner membrane before it enters the cytoplasm and the fluorescence is kept significantly low, hence *acrB*-knockout cells give a high fluorescent signal. The mutant was expressed equally to wild-type AcrB (Supplementary Figure 1). Figure 2A shows the growth curves panels for a selected concentration of the compounds (all concentrations can be seen in Supplementary Figure 2) and Figure 2B shows the ethidium bromide accumulation in wild-type and mutant AcrB. We found that the truncated mutant is a transporter which retains its exclusion ability for all tested compounds. The growth curves showing the vitality of the cells are in some concentrations (especially higher ones) just slightly lower than for wild-type AcrB (Supplementary Figure 2), but the mutant basically retains complete efflux efficiency compared to wild-type AcrB. The ethidium bromide exclusion assay also indicates that the truncated mutant is a functional transporter, able to pump ethidium out of the periplasm, with a fluorescent signal very close to wild-type AcrB. The deletion of three amino acids in this hoisting-loop segment of AcrB seems to have no effect on the function of the transporter.

### Crystal structure of the hoisting-loop truncation mutant AcrB

**Figure 3:**
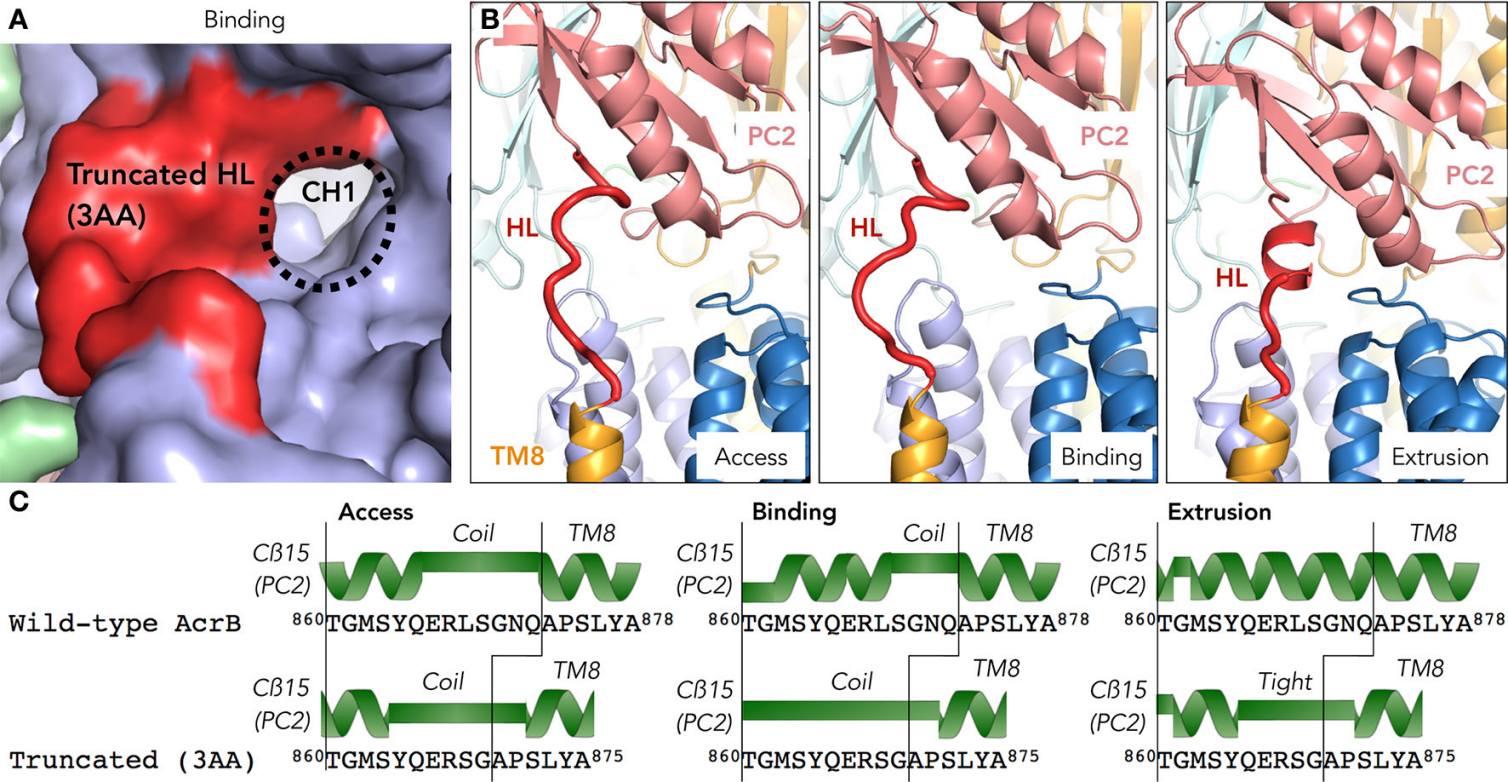
The crystal structure of a hoisting-loop truncation mutant AcrB. **(A)** Surface view representation of the binding monomer of the truncated mutant (3 amino acid deletion), zoomed in to the channel 1 (CH1) region. Although the loop (shown in red) is shortened, it still has an open channel. **(B)** A close-up view of the truncated hoisting-loop (dark red) in the access, binding and extrusion monomers in the crystal structure (3.0Å, Supplementary Table 1) of the 3 amino acid truncated AcrB mutant. **(C)** Secondary structure comparison of wild-type and truncated mutant AcrB of the hoisting-loop region from the Cß15 (PC2 domain) and the top of the TM8 in the access, binding and extrusion monomers. A random coil or stretched helix are depicted as a green bar and the helixes are depicted as a green helix depicted above the sequence. Abbreviations: CH1, channel 1; HL, hoisting-loop; TM8, transmembrane 8.

As the truncated mutant was a functional transporter, it raised the question how a loop-deletion mutant AcrB can still make the required conformational changes for the drug extrusion cycle. We were also speculating if the entrance of the surface channel CH1 was open or closed and thus whether or not the truncated loop would still be able to have a gating function for CH1. In order to address these questions, we solved the crystal structure of the hoisting-loop-truncated mutant AcrB at 3.0Å. The crystal was formed by hanging drop vapor diffusion at 25°C at reservoir pH 5.0 (see section Materials and Methods). In general, the structure is almost identical to wild-type AcrB (RMSD 0.70, 0.67 and 0.78Å for access, binding and extrusion monomers, respectively). Surprisingly, the structure shows that the entrance of CH1 to the proximal binding pocket is still completely open in the access and binding monomers (Figure 3A), similar to wild-type AcrB (Figure 3). The shortened loop flexibly retains the same length between Cβ5 and TM8 as in wild-type during the export cycle by unwinding a part of the α-helix (Figure 3C). Now, the random coil region is ^864^YQERSGAP^871^ in the access and binding monomers of the truncated mutant. Although the channel is completely open in the binding monomer, in the extrusion monomer, we found that the loop was tightened and stretched compared to wild-type AcrB. In contrast to wild-type AcrB, the loop was not an α-helix extending the top of TM8, but a stretched coil from the top of TM8 to the bottom of PC2 (Figures 3B,C). As seen in Figure 3C (which compares the secondary structure of wild-type and truncated AcrB), the top region of the TM8 helix became a coil rather than a helix in all monomers, indicating a shift: the random coil became slightly longer in the access and binding monomer and became a tight stretch in the extrusion monomer. The rest of the protein domains were unchanged compared to wild-type AcrB, indicating that the truncated loop was still able to act as a gate for CH1, facilitating efflux for certain compounds by opening the channel completely (Figure 3A), although the truncation of the loop did cause a significant tightening of the loop (Figure 3B). It also shows that this significantly moving region of AcrB is very flexible and that the deletion of three residues (about one turn of the α-helix) does not limit the ability of movement of the porter-region sub-domains, nor the energy transduction from the TM-domain to the porter-domain. A full comparison of the wild-type and truncated (3AA deletion) AcrB movement throughout the drug extrusion cycle can be seen in Supplementary Videos 1, 2 (cartoon and surface representation, respectively).

### Flexibility of the hoisting-loop segment

**Figure 4:**
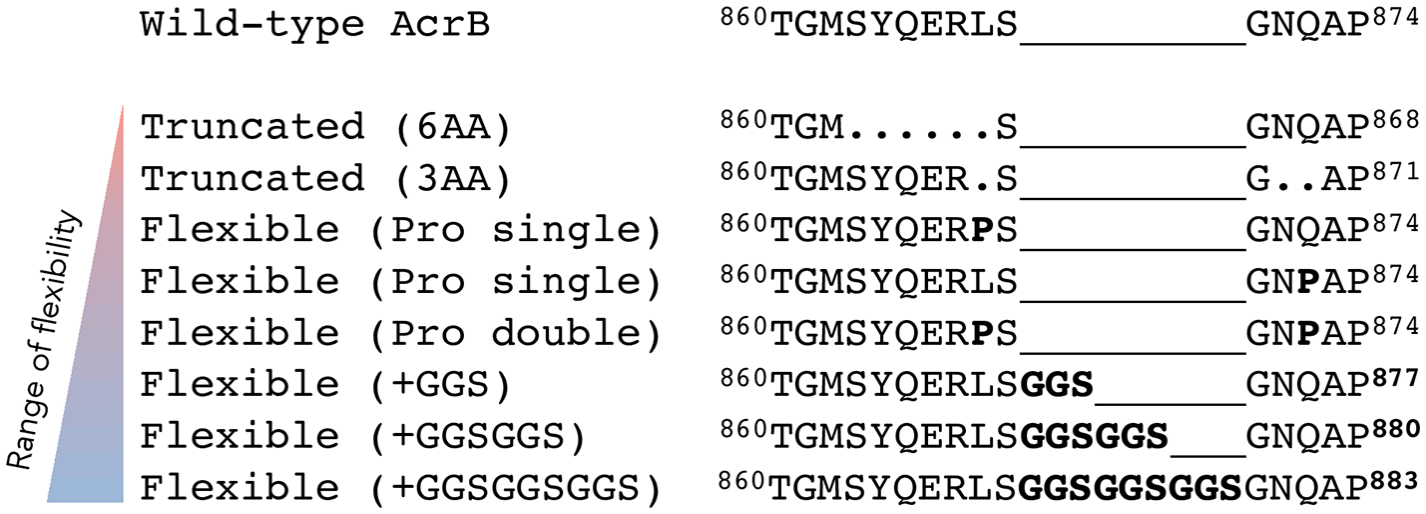
The range of flexibility of the hoisting-loop mutants. Sequence alignment of wild-type and hoisting-loop mutants of AcrB (here written as ^860^TGMSYQERLSGNQAP^874^). The triangle on the left indicates the degree of flexibility from low (red) to high (blue). “Truncated (6AA)” is the 6 amino acid deletion-mutant and “Truncated (3AA)” is the 3 amino acid deletion-mutant. Each “Pro Single” is either mutation L868P or Q872P, and “Pro Double” represents both mutations. The flexible elongated mutations are shown as “Flexible (+GGS),” “Flexible (+GGSGGS),” and “Flexible (+GGSGGSGGS).” A dot shows a deletion mutation and a line simply represents the relative difference compared to the most elongated flexible mutant in order to align all mutants.

The previous results indicate that a truncated mutant of AcrB is an active transporter and the protein adapts to a slightly different structural arrangement by undergoing compensatory conformational changes while maintaining full efflux ability. Now, we wanted to test the flexibility of this region more extensively. We created several more flexible and less flexible hoisting-loop mutants. First, we replaced the amino acid residues at both ends of this loop by proline residues (L868P and Q872P), as depicted in Figure 4. The proline mutations break the helix-formation and, as a result, the random coil structure would be fixed in the extrusion state. We created single and double mutants of these residue mutations. Furthermore, we created even more flexible mutants, by the introduction of repeats of GGS-residues: elongated mutant AcrB. The three elongated mutations were ^868^LSGGSGNQ, ^868^LSGGSGGSGNQ, and ^868^LSGGSGGSGGSGNQ (Figure 4). In addition, we constructed a hoisting-loop 6 amino acid truncated mutant, 3 amino acids more than the truncated mutant discussed previously, by deleting the six residues ^863^SYQERL^868^ from the loop (Figure 4). We tested all mutants (including the previously discussed truncated mutant) on efflux activity by determining the agar plate Minimal Inhibitory Concentration (MIC) values for nine compounds, which are shown in Table 1. We found that all proline mutants and the hoisting-loop (3AA deleted)-truncated mutant were fully functional. Also, the very flexible significantly elongated mutants with GGS-repeats were completely active. Even the 9AA elongated mutant is an active transporter, showing that the loop does not play an active role in the energy transduction. However, when the flexibility of the hoisting-loop was decreased too much (the 6AA deleted hoisting-loop mutant), the protein become mostly (but not entirely) inactive for all tested compounds. This shows that the flexibility of this region is a very important factor for the activity of the transporter. There is a limit in shortening the length of the hoisting-loop of about one turn of the helix (as discussed previously, see Figures 2, 3) and removing about two turns makes the efflux of compounds out of the cell very inefficient, probably by limiting the required movement of the PC2 domain. All mutants were expressed equally to wild-type AcrB (Supplementary Figure 1).

**Table 1:** Minimal Inhibitory Concentration (MIC) values for several structurally unrelated drugs.

**Strain**	**Flexibility**	**Minimal Inhibitory Concentration (MIC**, μ**g/mL)**								
		**CLX**	**BZK**	**EtBr**	**CV**	**NB**	**R6G**	**MINO**	**ACR**	**EM**
Δ*tolC*		0.25	4	8	2	2	32	0.5	8	2
*acrB*-KO		2	4	16	2	16	32	0.5	16	4
Wild-type AcrB		256	512	1,024	16	1,024	8,192	>2	1,024	128
Truncated (6 amino acids)	Lowest	16	32	256	8	128	256	0.5	32	32
Truncated (3 amino acids)		256	128	1,024	16	1,024	8,192	>2	1,024	128
Pro single (L868P)		256	128	1,024	16	1,024	8,192	>2	512	128
Pro single (Q872P)		256	256	1,024	16	1,024	8,192	>2	1,024	128
Pro double (L868P+Q872P)		256	512	1,024	16	1,024	8,192	>2	1,024	128
Elongated (+GGS)		512	512	>1,024	16	1,024	>8,192	>2	1,024	128
Elongated (+GGSGGS)		256	512	1,024	16	1,024	8,192	>2	1,024	128
Elongated (+GGSGGSGGS)	Highest	512	512	1,024	16	1,024	8,192	>2	1,024	128

*MIC concentrations were determined by step-wise dilutions gradients of the compounds in agar plates and the growth ability of MG1655ΔacrB E. coli cells (expressing the proteins from the plasmid) was determined. Values are in μg/mL. The colors indicate the degree of flexibility from low (red) to high (blue). Abbreviations: CLX, cloxacillin; BZK, benzalkonium; EtBr, ethidium bromide; CV, crystal violet; NB, novobiocin; R6G, rhodamine 6G; MINO, minocycline; ACR, acriflavine; EM, erythromycin*.

## Discussion

We show that the hoisting-loop is a highly flexible hinge that enables the large conformational changes of the subdomains of AcrB. Helix-breaking proline mutants, elongated loop mutants and truncated mutants show that this loop is extremely flexible. These protein mutants are still able to execute the required drug extrusion cycle (Table 1). The results of the truncated mutant indicate that the loop's conformational changes are a result of the movement of the protein during its extrusion process, and its flexibility enables these significant conformational changes in the AcrB drug transporter. The flexibility is indeed important for the protein function, as the 6 amino acid truncated mutant becomes mostly inactive (Table 1).

Crystal structures of AcrB TM-mutants by Eicher et al. (2012) support the hypothesis that there is a relationship between the TM-region and porter domain and between the conformational changes within the TM-domain and the configuration of certain amino acids within the proton relay pathway (Guan and Nakae, 2001; Su et al., 2006; Takatsuka and Nikaido, 2006; Eicher et al., 2014). In the access and binding monomers, Asp407, Asp408 (both TM4) and Lys940 (TM10) form ion pairs. In the extrusion monomer, the Lys940 is titled about 45° and the ion pairs are broken. In addition, residue Arg971 (TM11) changes its conformation significantly in the extrusion monomer compared to the other monomers (Murakami et al., 2006; Seeger et al., 2006; Sennhauser et al., 2007; Yamaguchi et al., 2015). In the binding monomer, Arg971 is separated from the cytoplasm by the residues Phe948, Met970 and Gln437, but open to the periplasm by a water channel (a center void). On the other hand, in the extrusion monomer, Arg971 is extended and exposed to a water channel connected to the cytoplasm, where it can supposedly release a proton to the interior of the cell. It is postulated that after the release of the proton from Arg971 to the cytoplasm, the residue takes a new proton from Asp408 and as a consequence, protonated Lys940 can restore its ion bond with Asp408, to form the access monomer. From this point, the proton of Lys940 is able to translocate to the deprotonated Asp408 and the proton from Asp407 can subsequently bind to Lys940, to form the binding monomer (Fischer and Kandt, 2011; Yamaguchi et al., 2015). Although the movements of the different regions and subdomains (such as TM2, TM8, 1α, the two TM-helix repeats, PN1/PC2 and PN2/PC2 and amino acids such as Lys940 and Arg971), as well as the protonation and deprotonation in the different stages of transport is well defined (Eicher et al., 2014) and possible mechanisms hypothesized (Eicher et al., 2014; Yamaguchi et al., 2015), it remains unclear how exactly the conformational coupling across the transmembrane and the porter domains occurs.

We found that the flexible hinge of the hoisting-loop helps facilitating the radical movements of the subdomains passively, as the elongated loop mutants (even the 9AA elongated mutant) exhibit the same phenotype as wild-type AcrB. As there are no other significant conformational changes in loops and regions linking the TM- and porter-domains directly during the drug extrusion cycle (e.g., similar to the hoisting-loop discussed in this study), another hypothesis needs to explain remote energy coupling mechanism of RND transporters. Computer simulations may shed a light on the complex mechanism of the exporter. Energy transduction might be due to the subtle changes in the TM-domain, explained by packing efficiency through the solvent-entropy effect translated to the porter domain, described by thermodynamics by computer simulations (Mishima et al., 2015) rather than a direct allosteric energy coupling. The structure of the AcrB trimer has often been compared to the αβ-subunits of bovine F_1_-ATPase, which also comprises similar functional access, binding and extrusion states by its monomers. Perhaps similar to F_1_-ATPase (Abrahams et al., 1994), the most stable conformation is disturbed (in this case by the binding of ATP instead of a proton) and the adjacent subunits react accordingly to these changes (compensating for energy loss) (Ito and Ikeguchi, 2015). This may be compared to the trimer reorganization of both the TM- and the porter-domain (the functionally rotating mechanism) and the interaction of the monomers with each other within the homotrimer (the entropy loss in one monomer due to the conformational changes during the export cycle is balanced by the entropy-gain of the other two monomers), facilitated by the necessary compensation of the protonation and deprotonation of AcrB (the proton-motive-force is necessary to energize the energy-unfavorable transitions during the export cycle) (Mishima et al., 2015). Although computer simulations can show the thermodynamic properties of the transporter during transport, they have their limitations and more biochemical and biophysical studies need to be done in order to elucidate the remote energy coupling mechanism of RND transporters. From our study, it is clear that the hoisting-loop region of AcrB plays an important role as facilitator between the transmembrane and porter domains, acting as a flexible hinge.

In summary, we show that the hoisting-loop is extremely flexible and necessary. Even a truncated loop mutant is able have a functional gating mechanism with an open CH1 channel, although additional truncation significantly inhibits the function of the exporter. Fully active elongated mutants show us that this region of AcrB is extremely flexible and that an increase of flexibility does not inhibit the energy transduction nor the function of the protein. The flexible loop can facilitate the significant changes in the porter subdomains PC1 and PC2. Our knowledge and understanding of RND transporters is crucial for the development of new antibiotic therapeutics.

## Author contributions

MZ and KH performed the molecular biological and biochemical experiments. KK, MZ, KH, RN, and KS performed the protein crystallization and structure analysis. KH and MZ designed the research. MZ, KN, and AY wrote the manuscript. The coordinates for the hoisting-loop mutant of AcrB have been deposited in the Protein Data Bank under accession number 5YIL.

### Conflict of interest statement

The authors declare that the research was conducted in the absence of any commercial or financial relationships that could be construed as a potential conflict of interest.
