# Supplementary material for: Hoisting-Loop in Bacterial Multidrug Exporter AcrB Is a Highly Flexible Hinge That Enables the Large Motion of the Subdomains
Source: Front Microbiol. 2017 Oct 25;8:2095. doi: 10.3389/fmicb.2017.02095 (PMC5661021; doi:10.3389/fmicb.2017.02095)
Supplement: Supplementary file 3 [file DataSheet1.DOCX]

Supplementary Material

Hoisting-loop in bacterial multidrug exporter AcrB is a highly flexible hinge that enables the large motion of the subdomains

**Martijn Zwama, Katsuhiko Hayashi, Keisuke Sakurai, Ryosuke Nakashima, Kimie Kitagawa, Kunihiko Nishino^*^, Akihito Yamaguchi***

# * Correspondence: Kunihiko Nishino. [nishino@sanken.osaka-u.ac.jp](mailto:nishino@sanken.osaka-u.ac.jp) Akihito Yamaguchi. [akihito@sanken.osaka-u.ac.jp](mailto:akihito@sanken.osaka-u.ac.jp)

**Supplementary Figures**


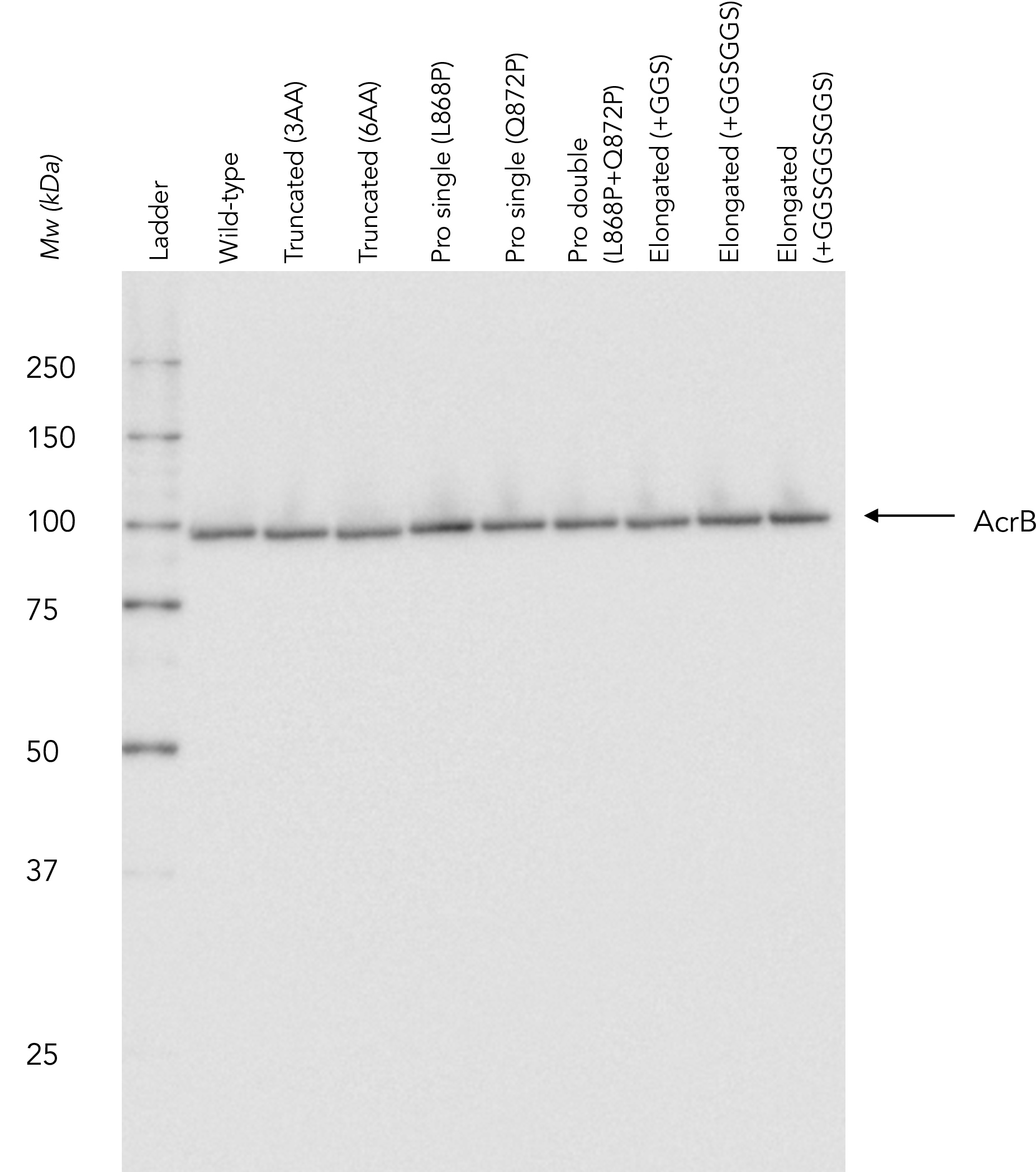


**Supplementary Figure 1. Expression levels of AcrB mutants.** Wild-type and mutant AcrB were expressed in MG1655∆*acrB* from pBAD33 plasmids. Membrane fractions of AcrB expressing cells were harvested and run on a 10% polyacrylamide gel (2µg protein) and transferred to a PVDF membrane. Western blotting was performed using an anti-polyhistidine antibody for the first reaction and mouse IgG HRP-linked antibody for the second reaction.

**
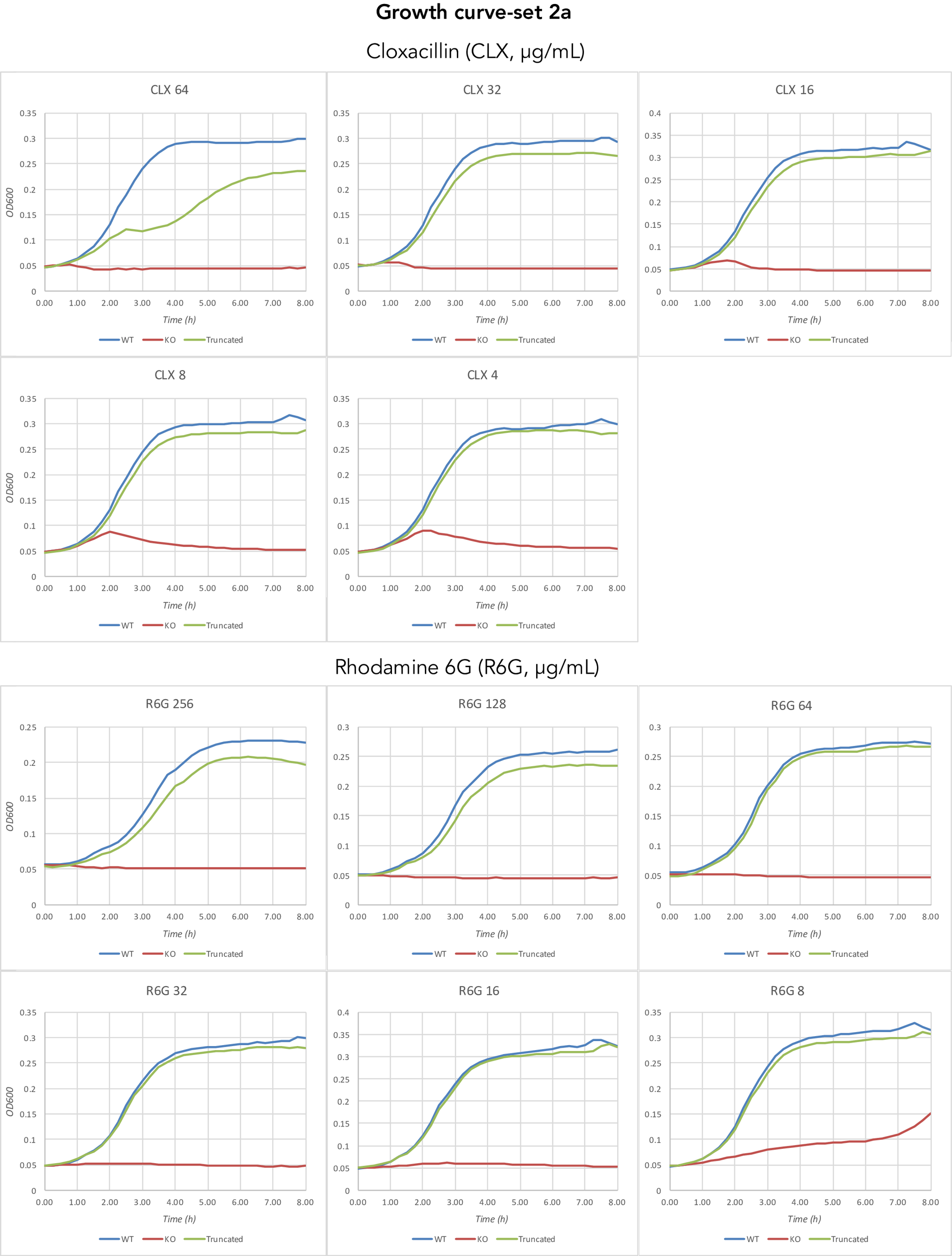
**

**
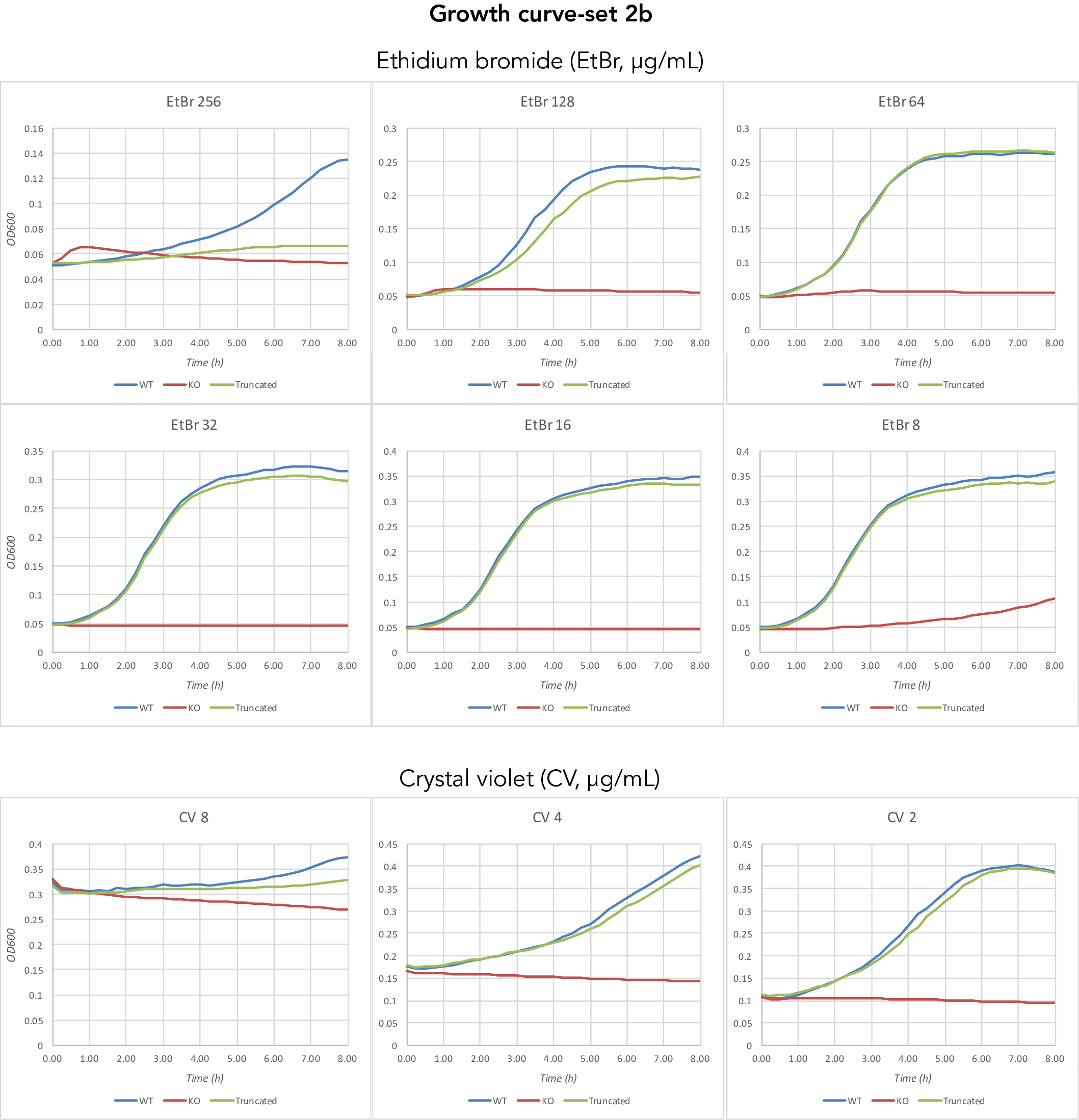

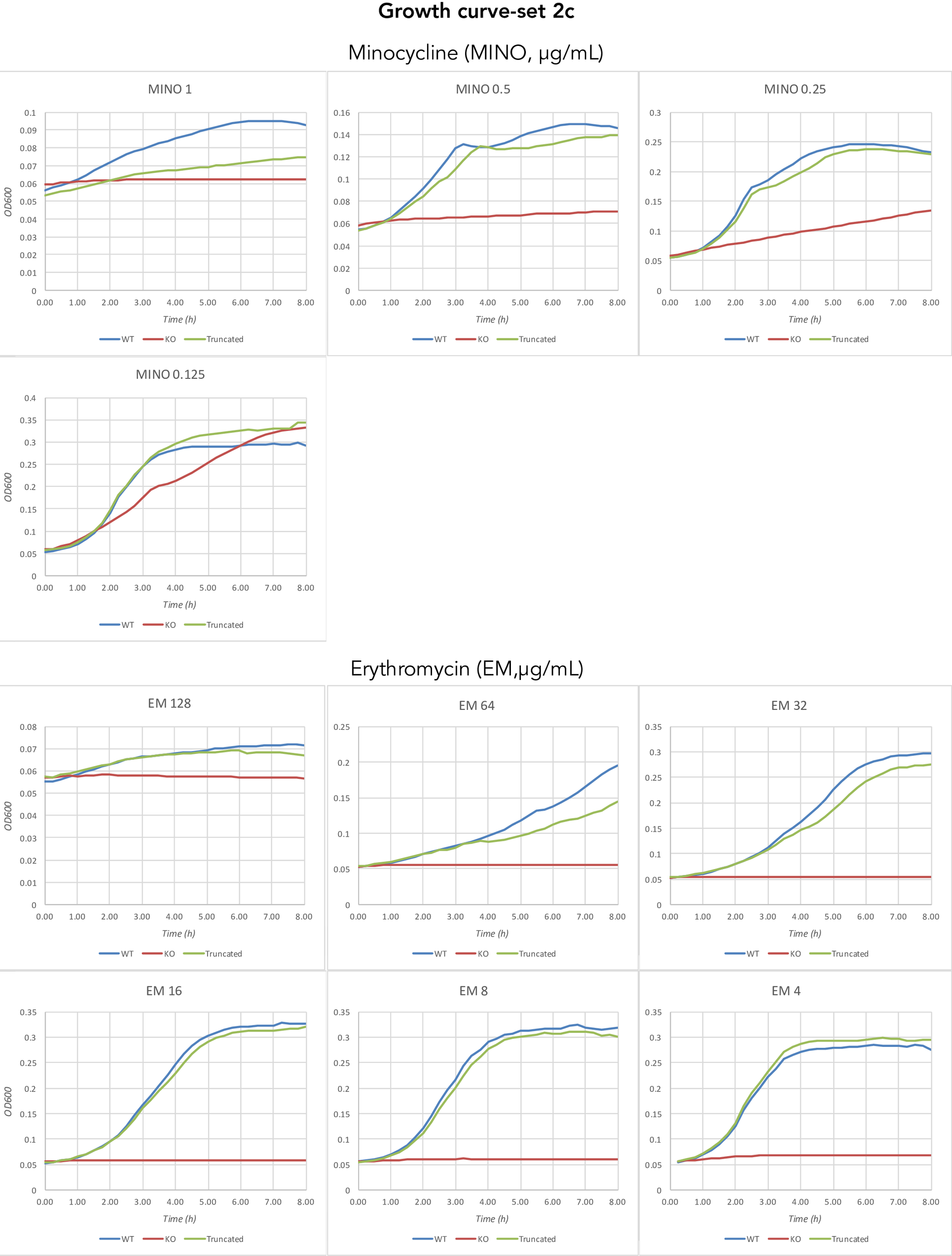

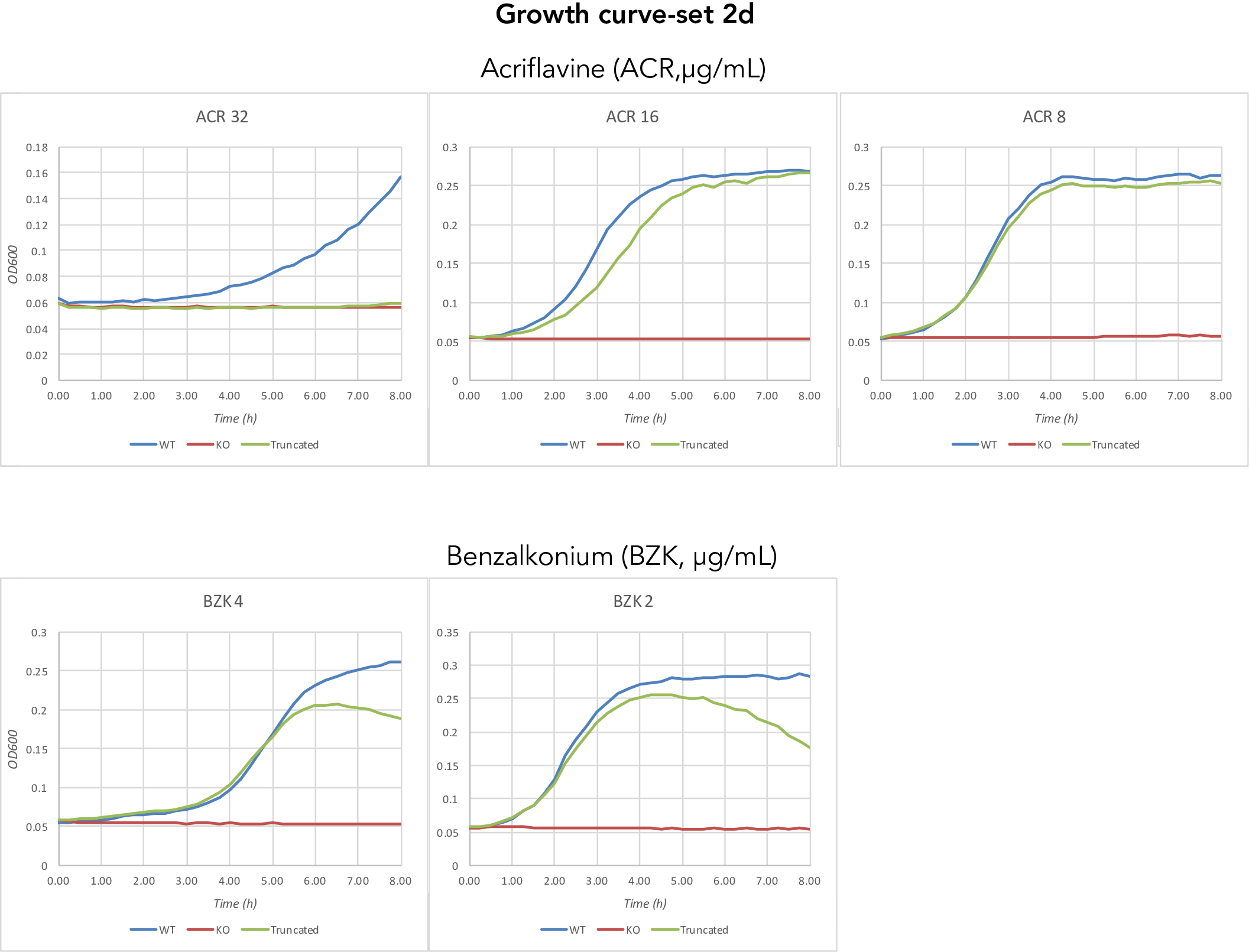
**

**Supplementary Figure 2. The effect of 3AA-truncated hoisting-loop mutations on the export activity of AcrB. (A-D)** Growth ability of *acrB*-knockout (KO, red), wild-type (WT, blue) and 3AA-truncated hoisting-loop mutant (truncated, green) AcrB-expressing cells. A 2-fold stepwise gradient of compounds was used. Concentrations are written above each panel in µg/mL. Shown is one of the results, repeats of the experiment gave similar results. Abbreviations: CLX, cloxacillin; BZK, benzalkonium; EtBr, ethidium bromide; CV, crystal violet; R6G, rhodamine 6G; MINO, minocycline; ACR, acriflavine; EM, erythromycin.

**Supplementary Tables**

**Supplementary Table 1. Data collection and refinement statistics for the AcrB HL-mutant.**

**
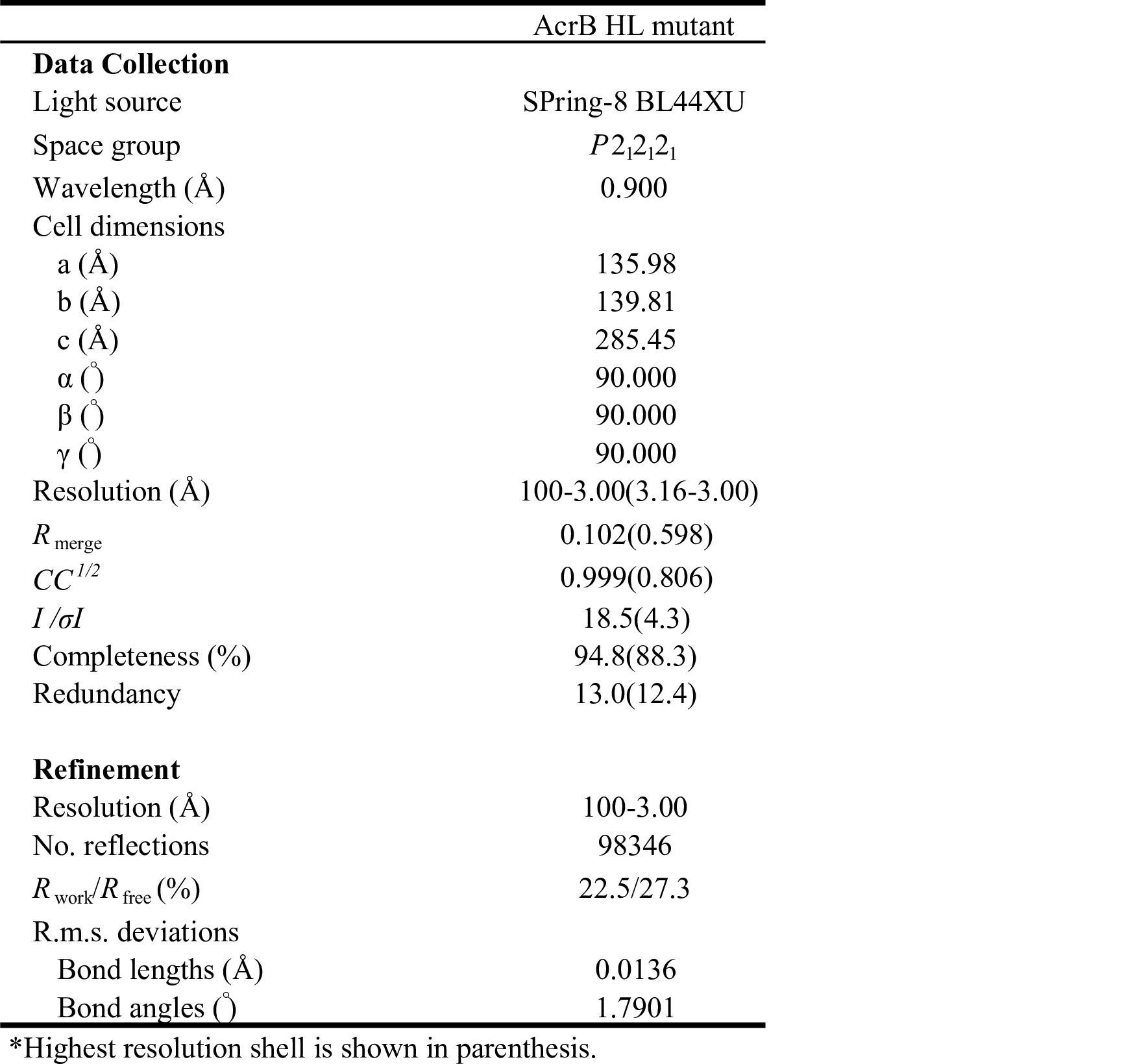
**

“AcrB HL mutant” represents the truncated 3 amino acid deletion mutant from the hoisting-loop of AcrB.

**Supplementary Videos**

**Supplementary Video 1. Conformational changes during the drug extrusion cycle in wild-type and truncated hoisting-loop mutant AcrB.** Shows are movements of the porter domains during the export cycle between the access, binding and extrusion stages. Left is wild-type and right is mutant AcrB. Red shows the hoisting-loop region. Crystal structure comparison can be found in Figure 3 and data analysis is given in Supplementary Table 1.

**Supplementary Video 2. Conformational changes and the opening and closing of CH1 during the export cycle in wild-type and mutant AcrB.** A surface-view of the movements of the porter region of wild-type (left) and truncated hoisting-loop mutant (right) AcrB. The hoisting-loop region is shown in red. The view is slightly upwards in order to see the opening and closing of the channel (CH1) in both wild-type and truncated AcrB.
